# QTL mapping of web blotch resistance in peanut by high-throughput genome-wide sequencing

**DOI:** 10.1186/s12870-020-02455-8

**Published:** 2020-06-03

**Authors:** Hua Liu, Ziqi Sun, Xinyou Zhang, Li Qin, Feiyan Qi, Zhenyu Wang, Pei Du, Jing Xu, Zhongxin Zhang, Suoyi Han, Shaojian Li, Meng Gao, Lina Zhang, Yujie Cheng, Zheng Zheng, Bingyan Huang, Wenzhao Dong

**Affiliations:** 1 College of Agronomy, Shenyang Agricultural University, Shenyang, 110866 PR China; 2 Industrial Crops Research Institute, Henan Academy of Agricultural Sciences / Key Laboratory of Oil Crops in Huang-Huai-Hai Plains, Ministry of Agriculture and Rural Affairs / Henan Provincial Key Laboratory for Genetic Improvement of Oil Crops, Zhengzhou, 450002 PR China; 3 Institute of Plant Protection, Henan Academy of Agricultural Sciences, Zhengzhou, 450002 PR China

**Keywords:** QTL mapping, Peanut, Web blotch resistance, Resequencing

## Abstract

**Background:**

Web blotch is one of the most important foliar diseases worldwide in peanut (*Arachis hypogaea* L.). The identification of quantitative trait loci (QTLs) for peanut web blotch resistance represents the basis for gene mining and the application of molecular breeding technologies.

**Results:**

In this study, a peanut recombinant inbred line (RIL) population was used to map QTLs for web blotch resistance based on high-throughput genome-wide sequencing. Frequency distributions of disease grade and disease index in five environments indicated wide phenotypic variations in response to web blotch among RILs. A high-density genetic map was constructed, containing 3634 bin markers distributed on 20 peanut linkage groups (LGs) with an average genetic distance of 0.5 cM. In total, eight QTLs were detected for peanut web blotch resistance in at least two environments, explaining from 2.8 to 15.1% of phenotypic variance. Two major QTLs *qWBRA04* and *qWBRA14* were detected in all five environments and were linked to 40 candidate genes encoding nucleotide-binding site leucine-rich repeat (NBS-LRR) or other proteins related to disease resistances.

**Conclusions:**

The results of this study provide a basis for breeding peanut cultivars with web blotch resistance.

## Background

Cultivated peanut (*Arachis hypogaea* L.) is one of the most important oil legumes in many countries. *A. hypogaea* is an allotetraploid (AABB, 2n = 4x = 40) with the genome size of ~ 2.7Gb [1]. The genome assembly results of two prominent parental cultivars of many Chinese peanut varieties “Fuhuasheng” and “Shitouqi” and one American cultivar “Tifrunner” were published in 2019 [2–4]. The assembled sequences were ~ 2.54 Gb and the predicated genes were about 60 ~ 80 million [2–4].

Peanut web blotch, also called muddy spot or net blotch [5, 6], caused by *Phoma araehidieola* Marasas, Pauer & Boerema [7, 8], is one of the most important foliar diseases in peanut. Peanut web blotch was first reported in Texas (U.S) in 1970s [9] and then, in the early 1980s, it was found in the major peanut growing areas of Shandong and Liaoning provinces of China [10, 11]. Subsequently, it was discovered in Shanxi province [12] and Henan province [13]. The web blotch disease can occur during the whole peanut growing period and can cause yield losses of 10% ~ 20% usually, but with the heaviest of more than 50%. Therefore, it has been regarded as one of the most urgent issues to be addressed in some peanut growing areas.

The fungus *P. araehidieola* pathogens normally overwinter within the crop residue and plants of all ages are susceptible [6]. Cloudy weather with frequent rains and temperatures (15–30 °C) favor fungal activity [14]. Spore germination and cuticle penetration occurred on leaflets of peanut and the symptoms appeared seven to 9 days after inoculation. The initial symptoms of small, irregular, brown to reddish brown lesions along the midrib of both young and old leaves were observed and the severe defoliation was observed in the infected fields of susceptible cultivars [15]. Following penetration of the cuticle, fungal hyphae grew just under the cuticle for a few mm beyond the point of penetration and ceased growth, which indicated that the defense responses of *P. araehidieola* infection is hypersensitive-type reaction [6].

Although the web blotch disease has considerable economic importance in peanut farming systems, previous researches on this disease are relatively few compared with those on other main peanut foliar diseases. Zhang et al. (2019) presented the draft genome sequence of a *Phoma araehidieola* isolate named Wb2 and indicated that the draft genome of Wb2 was about 34.11 Mb and contained 37,330 open reading frames (ORFs), with G + C content 49.23% [16]. Smith et al. (1979) reported that Virginia and runner market-type peanut cultivars are more resistant to web blotch than the Spanish market-type cultivars [17]. Zhang et al. (2011) showed that resistance to web blotch was controlled by three major genes and several minor genes, and identified one quantitative trait locus (QTL) located on linkage group (LG) 7 [18]. At present, researches on peanut web blotch mainly focus on the classification status of the pathogen asexual generation, the pathogen molecular biology, chemical control and epidemic rules [6–9, 14, 15]. A similar disease called net blotch, caused by *Pyrenophora teres* is also one of the major diseases in barley [19]. Many studies have identified a large region of chromosome 6H responsible for resistance to net blotch in barley [20–23] and a region in chromosome 3H harbors two predicted genes from the NBS-LRR gene family [24].

With advances in genomic sequencing technologies and the availability of diploid and tetraploid genome assemblies in *Arachis* species [1, 4], high-throughput genome-wide sequencing has become a primary strategy in peanut to identify single nucleotide polymorphisms (SNP) markers linked to resistance genes and QTLs. For example, four bacterial wilt resistance QTLs were identified on chromosome B02 using a RIL population and 2187 SNP markers [25]. A major QTL for resistance to late leaf spot, on chromosome B05, and two major QTLs for resistance to early leaf spot, on chromosomes A03 and B04, were mapped using a high-density genetic map comprising 2753 SNP markers and a F_9_ RIL population of 192 individual lines [26]. Additionally, 62 QTLs for 14 yield-related traits were detected on 12 chromosomes across three environments using a high-density genetic map including 2636 recombination bin markers and a F_6_ RIL population of 242 lines [27].

In the present study, high-throughput genome-wide sequencing technology was used to obtain SNP markers, and a SNP-based genetic linkage map was constructed to identify the QTLs for resistance to peanut web blotch. The results of this study will help to better understand the mechanisms of interaction between *P. araehidieola* and peanut and to develop resistant varieties.

## Results

### Evaluation of web blotch resistance

**Fig. 1:**
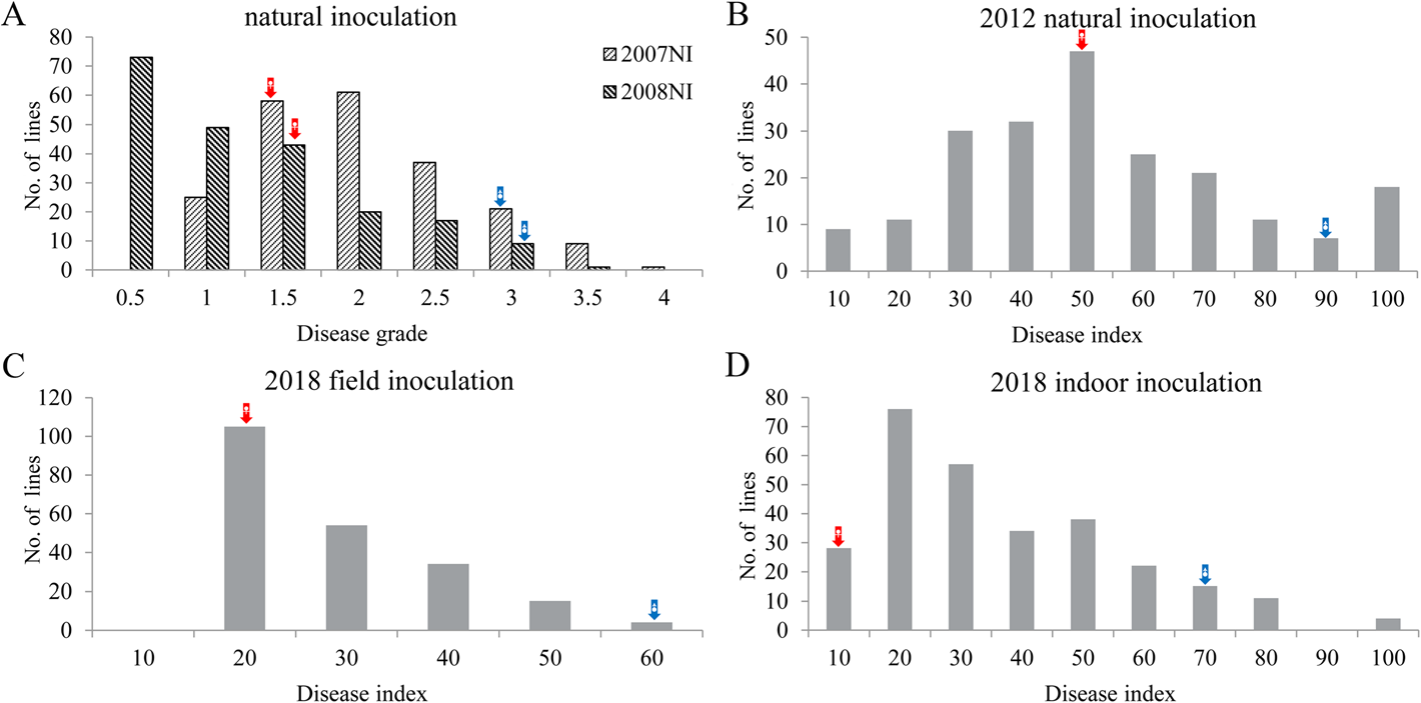
Response of RILs to peanut web blotch in five experimental trials. Red and blue arrows stand for the positons of resistant (female) and susceptible (male) parents respectively. **a**. The frequency distribution of disease grade under natural inoculation in the year 2007 and 2008. **b**. The frequency distribution of disease index under natural inoculation in the year 2012. **c**. The frequency distribution of disease index under field inoculation in the year 2018. **d**. The frequency distribution of disease index under indoor inoculation in the year 2018

Wide phenotypic variations in response to peanut web blotch were observed among RILs under all the five test environments in this study and the two parental varieties of the RIL population also showed significant difference in web blotch resistance (Fig. 1). The distributions of the disease scale recorded during the years 2007 and 2008 were shown in Fig. 1a, whereas the distributions of the disease index recorded during the years 2012 and 2018 (both in the field and indoor) were shown in Fig. 1b-d. The frequency distribution of disease index in the year 2012 was almost a normal distribution (Fig. 1b). The frequency distributions under other conditions displayed skewed distribution (Fig. 1a, c and d), which had no impact on the following mapping results because only the random error term of the phenotypic data was required to follow the normal distribution [28].

### Sequencing, SNP and bin markers discovery

A whole-genome resequencing strategy was applied to construct paired-end libraries for the parental lines and their 212 RIL progenies. The length of DNA fragments in the libraries was about 350 bp. Approximately, 490 Gb of clean data (Q20 > 96%) were produced, resulting from 6285 million reads, each with a length of 150 bp. In total, 600 million reads were generated for each of the two parents, whereas reads generated for each of the RILs varied from 22.37 to 24.83 million (Supplementary Table S1). Coverage rate, mapped reads rate, sequence depth and other results from alignment to the reference genome were shown in Supplementary Table S1. In particular, the coverage rate associated with the two parents Zheng8903 and Yuhua4 were 98.51 and 99.05%, respectively, whereas it ranged from 53 to 63.63% in the RIL population (Supplementary Table S1). The sequencing depth was 35.23× for both parents, whereas it ranged from 1.31× to 1.46× for the RIL population. Originally, 636,831 SNPs were called from the 214 samples using the GATK protocol. Then, 556,615 SNPs were retained after filtering for low quality loci in the two parents, due to missing values, heterozygosis, depth < 10 and GQ < 20. Finally, 138,039 SNPs which were homozygous and polymorphic between two parents were used for further analyses.

### Construction of physical recombination maps and high density genetic linkage map

To avoid errors caused by low coverage associated with RIL sequencing, a sliding window with 15 consecutive SNPs was used to find the more accurate recombined breakpoints. The physical recombination map of 212 RILs was constructed based on the recombination map of each progeny (Supplementary Figure S1). After that, all chromosomes of the 212 RILs were aligned and compared for the minimal of 100-kb intervals. As a result, a total of 3634 bin markers for the 212 lines were obtained in this way, and the genotypes and physical locations of the bins were given in Supplementary Table S2.

**Fig. 2:**
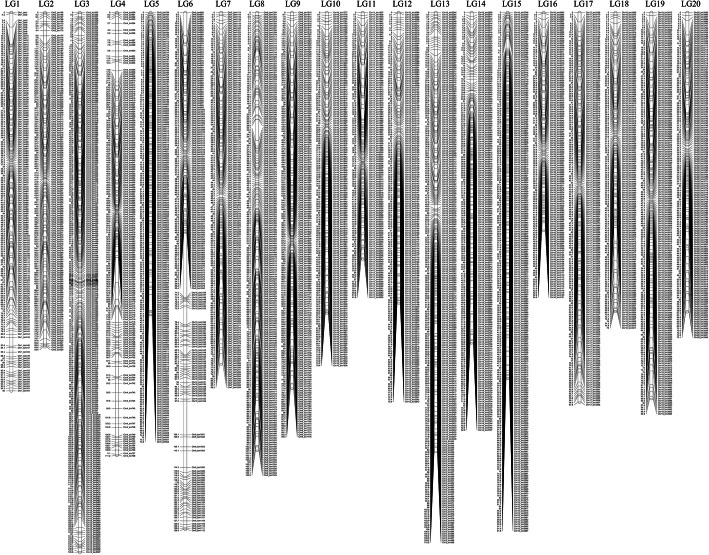
Genetic map obtained from the Zheng8903 × Yuhua4 RIL population

The obtained 3634 bin markers were used to construct a genetic linkage map by the software JoinMap**®**v5.0 [29]. Twenty linkage groups were generated and assigned to the 20 chromosomes of the cultivated peanut according to the physical positions. The total genome length was 1817.91 cM and the marker density across the 20 linkage groups ranged from 0.39 to 0.66 cM with an average of 0.50 cM (Table 1). The LG16 had the lowest marker number (129) and the shortest genetic length (54.58 cM), while LG3 had the highest marker number (277) and the longest genetic length (135.61 cM) (Table 1 and Fig. 2). More than 97.5% of the inter-markers distances were lower than 3 cM. The highest inter-marker distance (16.06 cM) was associated with LG6 (Table 1).

**Table 1:** Summary information on the 20 linkage groups (LGs) detected in this study

ID	Total markers	Total distance (cM)	Average distance (cM)	Max inter-marker distance (cM)	Inter-markers distances <= 3 cM
LG1	164	94.98	0.58	4.26	98.78%
LG2	152	83.88	0.55	2.27	100.00%
LG3	277	135.61	0.49	2.54	100.00%
LG4	168	111.21	0.66	6.33	98.21%
LG5	194	75.76	0.39	3.96	99.48%
LG6	200	129.82	0.65	16.06	97.50%
LG7	170	88.20	0.52	2.54	100.00%
LG8	210	109.86	0.52	5.35	99.52%
LG9	192	94.31	0.49	2.82	100.00%
LG10	160	75.41	0.47	1.74	100.00%
LG11	129	61.81	0.48	3.39	99.22%
LG12	176	72.90	0.41	1.74	100.00%
LG13	240	110.15	0.46	2.41	100.00%
LG14	189	82.88	0.44	2.14	100.00%
LG15	234	91.81	0.39	2.41	100.00%
LG16	129	54.58	0.42	2.27	100.00%
LG17	178	97.78	0.55	2.68	100.00%
LG18	143	74.99	0.52	3.39	98.60%
LG19	182	96.90	0.53	3.39	98.90%
LG20	147	75.07	0.51	3.67	99.32%
Total	3634	1817.91	0.50

### QTL mapping and candidate genes prediction for peanut web blotch resistance

**Fig. 3:**
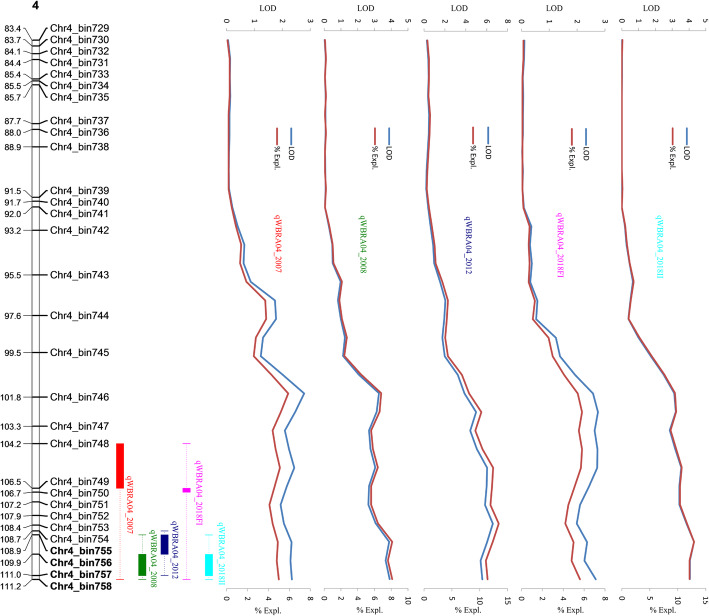
Position, LOD and PVE (%Expl.) curves of the QTL detected on LG4

**Fig. 4:**
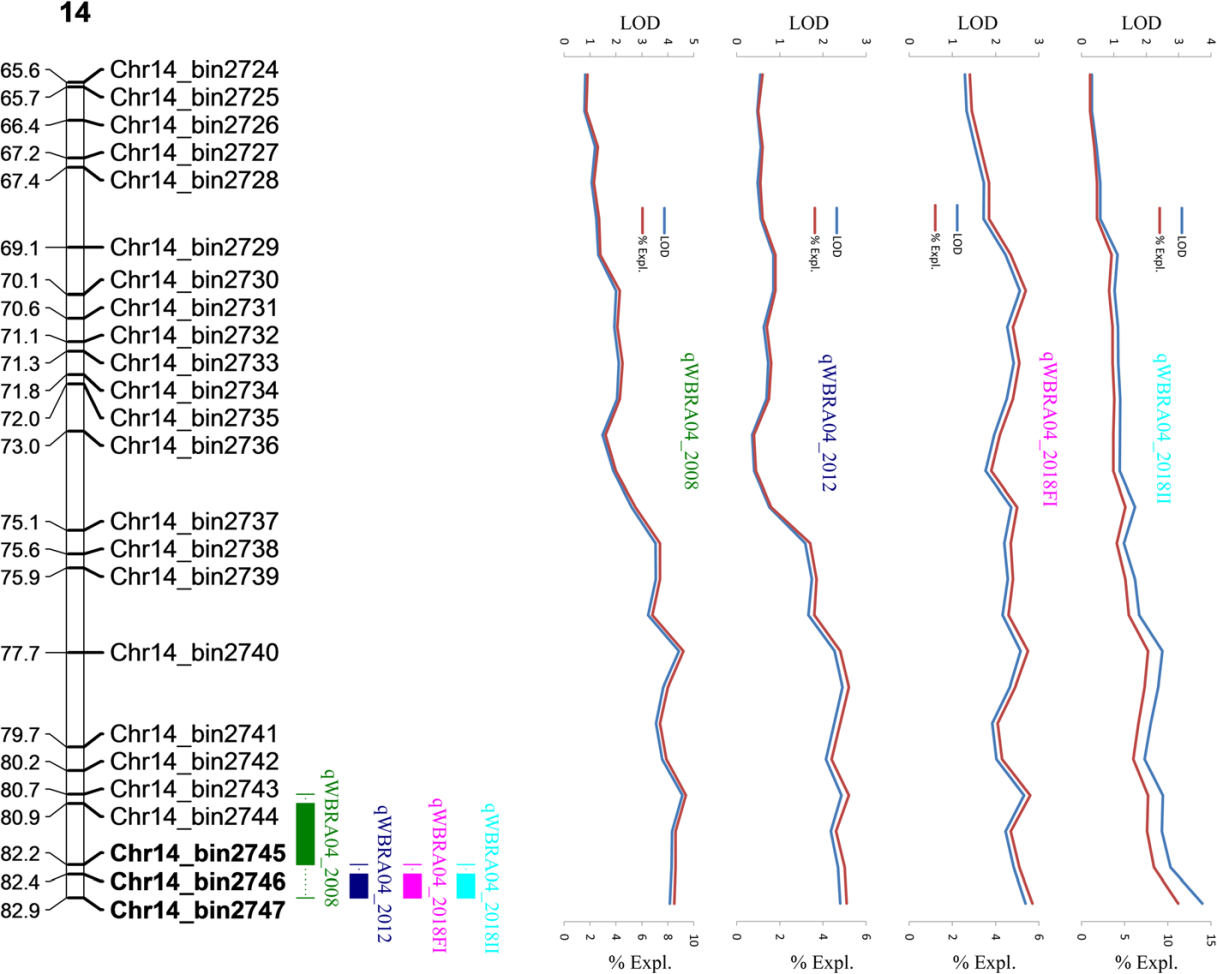
Position, LOD and PVE (%Expl.) curves of the QTL detected on LG14

QTL mapping of peanut web blotch resistance was performed with MapQTL**®** v6.0 [30], using phenotypic data collected across five environments. Eight QTLs associated with peanut web blotch resistance, located in eight different LGs, were confirmed in at least two environments, explaining from 2.8 to 15.1% of phenotypic variation and displaying LOD values ranging from 1.32 to 7.45 (Table 2). Two QTLs (*qWBRA04* and *qWBRA14*) located on LG04 and LG14 were significantly associated with resistance in all the five testing environments in this study (Table 2, Fig. 3 and Fig. 4) and explained more than 10% of phenotypic variation, indicating they are probably the major QTLs with stable expression. Except for *qWBRA13* and *qWBRA05*, which were detected in four and two environments respectively, the other four QTLs *qWBRA03*, *qWBRA16*, *qWBRA17*, *qWBRA19* were detected in three environments (Table 2). Absolute values displayed by the additive effect ranged from 0.11 to 8.29, and were negative for the QTLs on LG4, LG5, LG13, LG14, LG19 (indicating that the favorable alleles originated from the resistant parent Zheng8903), and positive for the QTLs on LG3, LG16 and LG17 (indicating that the favorable alleles originated from the susceptible parent Yuhua4).

**Table 2:** Quantitative trait loci (QTLs) for peanut web blotch resistance identified in this study

QTL	LG	Position (cM)	Left-Right Marker	LOD	PVE%	Add effects	Environment
*qWBRA03*	LG03	29.84	Chr3_bin366-Chr3_bin367	3.48 ~ 5.37	7.4 ~ 11.0	0.21 ~ 5.85	2007, 2008, 2012
*qWBRA04*	LG04	110.17	Chr4_bin756-Chr4_bin757	2.35 ~ 7.45	5.0 ~ 15.1	−8.29 ~ −0.15	2007, 2008, 2012, 2018^a^, 2018^b^
*qWBRA05*	LG05	0.48	Chr5_bin760-Chr5_bin761	2.88 ~ 3.28	6.9 ~ 8.8	−6.1 ~ −5.61	2012, 2018^b^
*qWBRA13*	LG13	64.06	Chr13_bin2385-Chr13_bin2386	1.88 ~ 3.86	4.0 ~ 8.0	−5.32 ~ −0.15	2007, 2008, 2012, 2018^a^
*qWBRA14*	LG14	82.88	Chr14_bin2746-Chr14_bin2747	1.32 ~ 4.08	2.8 ~ 11.2	−6.9 ~ −.011	2007, 2008, 2012, 2018^a^, 2018^b^
*qWBRA16*	LG16	41.84	Chr16_bin3084-Chr16_bin3085	2.16 ~ 3.18	5.5 ~ 6.7	0.18 ~ 5.51	2008, 2012, 2018^b^
*qWBRA17*	LG17	45.25	Chr17_bin3253-Chr17_bin3254	3.33 ~ 4.98	7.0 ~ 10.2	0.21 ~ 6.83	2008, 2012, 2018^a^
*qWBRA19*	LG19	94.09	Chr19_bin3690-Chr19_bin3691	1.93 ~ 3.03	4.1 ~ 6.4	−2.22 ~ −0.13	2007, 2008, 2018^a^

2018^a^stands for field, 2018^b^ stands for indoor

To identify candidate genes for peanut web blotch resistance, coding sequences in the genomic region associated with the QTLs *qWBRA04* and *qWBRA14* were examined for predicted function, according to the *Arachis hypogaea* cv. Tifrunner reference genome annotation database [4]. A total of 40 candidate genes were identified with a putative role in disease resistance (Table 3). In detail, the region of *qWBRA04*, spanning a linkage interval of 1.10 cM, corresponds to a physical interval of ~ 86 kb and contains four nucleotide binding site-leucine rich repeat (NBS-LRR) genes. The genes *Arahy.Q7VTCQ* and *Arahy.9YX67Z* contain coiled-coil (CC) domains and the genes *Arahy.SK6LYR* and *Arahy.1RZ0PJ* contain Toll-interleukin receptor (TIR) domain (Table 3). The region of *qWBRA14*, spanning 0.48 cM, physically corresponds to ~ 2.8 Mb and contains 37 genes encoding disease resistance protein. Among them, 19 genes contain TIR domains and one gene contains CC domain, whereas the remaining 17 genes encode other proteins with a putative role in disease resistance, such as a *bZIP* transcription factor and a *WRKY* transcription factor-like protein.

**Table 3:** Candidate genes for peanut web blotch resistance in the region of *qWBRA04* and *qWBRA14*

Gene model	Gene Location	Annotation
Arahy.Q7VTCQ	Arahy.04:125904648–125,913,229	CC-NBS-LRR
Arahy.SK6LYR	Arahy.04:125919867–125,922,353	TIR-NBS-LRR
Arahy.9YX67Z	Arahy.04:125933796–125,935,754	CC-NBS-LRR
Arahy.1RZ0PJ	Arahy.04:125978283–125,982,796	TIR-NBS-LRR
Arahy.MR7539	Arahy.14:140429517–140,433,390	TIR-NBS-LRR
Arahy.5JS28N	Arahy.14:140673363–140,675,200	TIR-NBS-LRR
Arahy.098P1D	Arahy.14:140716472–140,720,198	TIR-NBS-LRR
Arahy.B7IYCZ	Arahy.14:140737488–140,743,003	TIR-NBS-LRR
Arahy.GQ7H4X	Arahy.14:140748678–140,752,470	TIR-NBS-LRR
Arahy.KRT76W	Arahy.14:140775690–140,779,279	TIR-NBS-LRR
Arahy.ARVN7W	Arahy.14:140932228–140,936,295	Ethylene-responsive transcription factor 1B
Arahy.QC7JPT	Arahy.14:140964016–140,967,594	MYB/SANT-like DNA-binding domain protein
Arahy.ULYV8E	Arahy.14:141015920–141,031,728	E3 ubiquitin protein ligase DRIP2-like
Arahy.M6XMZZ	Arahy.14:141138899–141,141,424	Disease resistance protein
Arahy.GF38LY	Arahy.14:141242234–141,244,304	TIR-NBS-LRR
Arahy.Z4PSSR	Arahy.14:141280146–141,281,153	TIR-NBS-LRR
Arahy.38PKYC	Arahy.14:141292017–141,295,699	TIR-NBS-LRR
Arahy.3HI1A9	Arahy.14:141393957–141,398,159	TIR-NBS-LRR
Arahy.6V6NN7	Arahy.14:141400695–141,406,643	TIR-NBS-LRR
Arahy.1B47VV	Arahy.14:141409110–141,411,145	LRR and NB-ARC domain disease resistance protein
Arahy.UDNB1T	Arahy.14:141411916–141,413,951	LRR and NB-ARC domain disease resistance protein
Arahy.1VN7JI	Arahy.14:141416418–141,422,328	TIR-NBS-LRR
Arahy.D05D8X	Arahy.14:141438610–141,439,200	Disease resistance protein RPP13-like
Arahy.5TMH4E	Arahy.14:141431509–141,437,763	TIR-NBS-LRR
Arahy.NA6KD0	Arahy.14:141445227–141,450,684	TIR-NBS-LRR
Arahy.WD8J75	Arahy.14:141458141–141,462,928	TIR-NBS-LRR
Arahy.UZFH7Q	Arahy.14:141463237–141,467,397	TIR-NBS-LRR
Arahy.5MNM78	Arahy.14:141468339–141,471,341	TIR-NBS-LRR
Arahy.388Y5C	Arahy.14:141508791–141,512,359	TIR-NBS-LRR
Arahy.M9HGBQ	Arahy.14:141514206–141,520,849	bZIP transcription factor
Arahy.BJT98M	Arahy.14:141539637–141,544,390	WRKY transcription factor-like protein
Arahy.EDAK6K	Arahy.14:141674291–141,674,683	CC-NBS-LRR
Arahy.RZ4S3T	Arahy.14:141672176–141,674,272	Disease resistance protein
Arahy.QH59CH	Arahy.14:141683064–141,685,622	Disease resistance protein
Arahy.GM4B0I	Arahy.14:141689106–141,691,574	Disease resistance protein
Arahy.U06TWT	Arahy.14:141696720–141,699,293	Disease resistance protein
Arahy.8I61S0	Arahy.14:141708397–141,711,939	Disease resistance protein
Arahy.ZY30Q0	Arahy.14:141719033–141,719,888	Disease resistance protein
Arahy.N7WF48	Arahy.14:141789316–141,794,004	TCP7 transcription factor
Arahy.QA8QB9	Arahy.14:143064164–143,068,579	E3 ubiquitin-protein ligase

To further validate the candidate genes, the mutation type of the SNPs located in the candidate genes were analyzed (Table 4). There were 24 polymorphic SNPs between two parents located in seven genes, eight of which were in noncoding DNA regions and two of which were synonymous mutation, and the remained 14 SNPs on chromosome A03, A04, and A14 were non-synonymous. The 14 non-synonymous SNPs were located in four coding sequence regions, four of which were in the region of *Arahy.LFE0TK*, one in *Arahy.Q7VTCQ*, three in *Arahy.1RZ0PJ* and six in *Arahy.MR7539.* All of the four genes were NBS-LRR, and three of them contained TIR domain except that *Arahy.Q7VTCQ* contained CC domain.

### KASP design and validation

To realize molecular marker assisted breeding for web blotch resistant peanut varieties, the 24 polymorphic SNPs between two parents (Table 4) were used to develop KASP (Kompetitive allele specific PCR) markers which were validated using 47 lines (23 resistant lines and 24 susceptible lines) from this population. Results indicated that three SNPs on chromosome A04 in the region of *Arahy.Q7VTCQ* might be related to peanut web blotch resistance (Supplementary Table S3). Most of the tested lines (42 out of 47 lines) showed consistency between their phenotypes and genotypes in different test conditions. Two susceptible genotypes (WB6235 and WB6360) that showed low level of infection in some conditions, probably escaped from inoculation (Supplementary Table S3). One susceptible line (WB6204) and two resistant lines (WB6248 and WB6367) were inconsistent with their phenotypes (Supplementary Table S3).

## Discussion

A high density genetic linkage map with 3634 bin markers was constructed based on high-throughput whole-genome sequencing and a sliding window strategy. The order of the markers on the genetic map was overall consistent with the physical order according to the peanut genome assembly, except for some translocation occurring between LG3 and LG13 and between LG6 and LG16. A total of 277 bin markers were distributed on LG3, while the physical positions of the last 70 markers were on chromosome A13, and the physical positions of the last 47 markers of LG13 were on chromosome A03. Moreover, 43 bin markers from chromosome A16 were inserted in the LG6. The reason for such seeming translocation might be that there were some assembly errors in the reference genome which were illustrated in Peanutbase (https://www.peanutbase.org/data/public/Arachis_hypogaea/Tifrunner.gnm1.KYV3/) [31]. The genetic orders of the markers on LG1, LG4, LG5, LG10, LG12 and LG18 were fully consistent with their physical orders and a few markers on the other LGs were inversed with the adjacent markers.

In total, eight novel QTLs distributed on eight chromosomes were identified for peanut web blotch resistance, which were confirmed in at least two of the experimental trials. Three of them were major QTLs, as they were associated with phenotypic variance explained (PVE) > 10%, whereas the other five were minor QTLs, in accordance with the predicted results of Zhang et al. (2011). Except for *qWBRA03*, which was detected only in naturally conditions, the other seven QTLs were detected both in the natural and artificial inoculation conditions. The two major QTLs *qWBRA04* and *qWBRA14* were stably detected across all the five testing environments in this study, and thus might be of great potential in breeding resistant peanut varieties.

In the present study, we identified 55 candidate disease resistant genes in the target region of eight QTLs (Tables 3 and 5), of which 40 were linked with the two major and stable QTLs *qWBRA04* and *qWBRA14* (Table 3), while the other 15 were associated with the other six QTL intervals (Table 5). The 40 candidate genes covered by the intervals of *qWBRA04* and *qWBRA14* included 21 TIR-NBS-LRR and 3 CC-NBS-LRR, which are the two well-known *R* gene types [32]. Also, there were two candidate genes encoded LRR and NB-ARC (nucleotide-binding domain shared with APAF-1, various R-proteins and CED-4) domain, which also encoded resistance genes [33]. Among the remained 15 genes, seven of them function in the downstream pathways of resistant signaling, the other eight encoded proteins contain disease resistance response related domains but could not assigned to the well-known R-gene types (Table 3).

**Table 4:** The mutation type of the SNPs located in the candidate genes

Gene model	Chromosome	Position	Resistant parent allele	Susceptible parent allele	Mution type
Arahy.LFE0TK	Arahy.03	5,689,199	A	T	noncoding DNA regions
	Arahy.03	5,689,634	T	C	T → C, Glu → Gly
	Arahy.03	5,692,237	A	G	A → G, Cys → Arg
	Arahy.03	5,694,410	C	T	C → T, Arg → His
	Arahy.03	5,696,415	A	C	A → C, Ser → Ala
Arahy.Q7VTCQ	Arahy.04	125,905,992	T	C	noncoding DNA regions
	Arahy.04	125,907,949	A	G	noncoding DNA regions
	Arahy.04	125,909,638	C	T	noncoding DNA regions
	Arahy.04	125,911,313	G	T	Synonymous mutation, Ala
	Arahy.04	125,912,343	G	T	G → T, Asp→Tyr
Arahy.1RZ0PJ	Arahy.04	125,980,437	C	G	C → G, Thr → Ser
	Arahy.04	125,981,121	A	C	A → C, Lys → Thr
	Arahy.04	125,981,989	A	C	A → C, Lys → Asp
Arahy.00XS3D	Arahy.05	7,865,324	T	G	noncoding DNA regions
Arahy.IY6P5S	Arahy.05	8,315,940	T	C	noncoding DNA regions
Arahy.MR7539	Arahy.14	140,432,094	G	C	G → C, Lys → Asp
	Arahy.14	140,432,405	T	A	T → A, Phe → Tyr
	Arahy.14	140,432,499	T	G	Synonymous mutation, Val
	Arahy.14	140,432,542	G	C	G → C, Val → Leu
	Arahy.14	140,432,546	T	G	T → G, Val → Gly
	Arahy.14	140,432,725	G	T	G → T, Gly → Cys
	Arahy.14	140,432,848	G	C	G → C, Glu → Gln
Arahy.NC1Z37	Arahy.17	26,849,446	C	T	noncoding DNA regions
	Arahy.17	26,855,030	C	T	noncoding DNA regions

**Table 5:** Candidate genes for peanut web blotch resistance in the region of six QTLs identified in this study

Gene model	Gene Location	Annotation
Arahy.LFE0TK	Arahy.03:5688642–5,697,084 (− strand)	TIR-NBS-LRR
Arahy.25H20J	Arahy.03:5707178–5,707,681 (− strand)	TIR-NBS-LRR
Arahy.FHP2K2	Arahy.03:5719557–5,720,120 (+ strand)	Disease resistance-responsive protein
Arahy.PQJ7DP	Arahy.03:5791188–5,792,666 (+ strand)	MYB transcription factor
Arahy.00XS3D	Arahy.05:7862831–7,866,802 (+ strand)	Ethylene-responsive transcription factor 3
Arahy.S3U17M	Arahy.05:8182461–8,184,440 (− strand)	MYB family transcription factor
Arahy.90DV49	Arahy.05:8258615–8,262,245 (+ strand)	Transcription factor SPATULA-like(bHLH)
Arahy.IY6P5S	Arahy.05:8314264–8,318,032 (+ strand)	Protein kinase superfamily protein
Arahy.DIZB8V	Arahy.13:43668419–43,670,419 (+ strand)	Ethylene-responsive transcription factor 3
Arahy.0E1GBK	Arahy.13:44877348–44,878,744 (− strand)	ZIP zinc/iron transport family protein
Arahy.8JT992	Arahy.13:45354167–45,355,323 (− strand)	MYB transcription factor
Arahy.2NFP2H	Arahy.19:157562932–157,564,981 (+ strand)	Heat shock transcription factor A2
Arahy.5KF5UG	Arahy.19:157602650–157,604,922 (− strand)	bZIP transcription factor family protein
Arahy.ZMEP2D	Arahy.16:121662983–121,668,124 (+ strand)	HSP20-like chaperones superfamily protein
Arahy.NC1Z37	Arahy.17:26845330–26,857,334 (− strand)	bZIP transcription factor family protein

A total of 26 NBS-LRR genes were identified to be related to peanut web blotch resistance in this study. NBS-LRR is the biggest category of R genes [34] and has been identified at the genome-wide level in *Arachis* [35]. It had been found that NBS-LRRs were involved in response to late leaf spot, tomato spotted wilt virus, and bacterial wilt in *A. duranensis*, *A. ipaensis*, and *A. hypogaea* [36]. It could be concluded that NBS-LRR was also involved in the resistance to peanut web blotch, but the regulatory mechanism in the process of disease resistance needs to be further studied.

The results of three validated KASP markers indicated that the gene *Arahy.Q7VTCQ* (CC-NBS-LRR) might be one of the resistant genes for peanut web blotch in a great possibility. The reason of the inconsistence between the phenotypes and genotypes of three test lines may be that the three KASP markers were not completely linked with peanut web blotch resistance. Therefore, further study will be needed to design closer linked markers to be employed in molecular marker assisted breeding (MAS).

## Conclusion

In this study, eight QTLs for peanut web blotch resistance were detected and two major QTLs *qWBRA04* and *qWBRA04* were linked to 40 candidate genes encoding NBS-LRR or other proteins related to disease resistance, which may shed some insights on understanding web blotch resistance and facilitate the development of resistant peanut cultivars.

## Methods

### Plant materials

A RIL population consisting of 212 F_12_ lines derived from the parental cross combination between lines Zheng8903 and cultivar Yuhua4 was used in this study. The female parent Zheng8903 with the pedigree ‘79–266//71–31/Chico’ is a breeding line showing high level of resistance to peanut web blotch [37]. The male parent Yuhua4 is a variety released in 1991 by the Henan Academy of Agricultural Sciences and we obtained the seeds from our own inventory [38], showing high level of susceptibility to web blotch [37]. All the plant materials including RILs and its parents mentioned above were developed and preserved in the corresponding author’s lab.

### Experimental trials and phenotyping

All the experimental trials to evaluate response to peanut web blotch were conducted at the research station of the Henan Academy of Agricultural Science from May to September. Plant materials mentioned above were evaluated following natural infection, in field trials carried out in 2007, 2008 and 2012, and following artificial inoculation, in field and indoor trials carried out in 2018. For field trails, twenty seeds for each genotype were sown in 3 m long and 0.4 m wide plots, according to a complete random block design with two replicates for natural infection and three replicates for artificial inoculation. For natural infection in the year 2007, 2008 and 2012, disease evaluation was carried out before harvest according to the 0–4 scale described in Yuan et al. (2004) [39]. For field inoculation in the year 2018, plots were sprayed with an inoculum of 1.6 × 10^− 3^ g/ml at the flowering stage [40] and disease evaluation was carried out 20 days after inoculation according to the 0–9 scale described in Yu (2011) [41]. For indoor inoculation, five plants were inoculated for each line with two replications at the 6 leaves stage. The inoculum concentration was 2 × 10^6^ conidia/ml and the preparation of conidia suspension described by Zhang (2019) [37] was followed. Two weeks after inoculation, 12 inoculated leaves at the main stem were collected and the lesion area for each was scanned by the Leaf Area Meter (Wanshen LA-S). The indoor classification standard of peanut web blotch was as follows: for scale 0, no lesion detected; scale 1, 0<lesion area<6%; scale 2, 6% ≤ lesion area<25%; scale 3, 25% ≤ lesion area<50%; scale 4, 50% ≤ lesion area<75%; scale 5, 75% ≤ lesion area.

### Sequencing and genotyping

Genomic DNA of the parents and 212 RILs was extracted from young leaf tissues using the Plant genome DNA extraction kit (TIANGEN) and randomly sheared by sonication. The DNA fragments with the length of 300 bp were recovered by electrophoresis. After ligating DNA fragments with adapters, libraries were paired-end sequenced using the Illumina Hi-seq platform with read length of 150 bp.

After trimming adapters and low quality reads, clean data was used for aligning to the reference genome, allowing SNP identification and genotyping. In detail, the assembly of *Arachis hypogaea* cv. Tifrunner was used as the reference genome [4]. The aln command in the software bwa-0.7.10 was used to align clean data to the reference genome, and unique reads were used for subsequent SNP variation detection by the software GATK3.3.0. The obtained SNP sets of two parents were filtered used the missing values, heterozygosis, depth and GQ value and the homozygous and polymorphic loci were used for the RIL population. The binary alignment mapping (BAM) files obtained in this study have been submitted to the BioProject database at NCBI under the BioProject ID: PRJNA602098.

### Linkage map construction and QTL mapping

As the accuracy of call at single SNP loci was low, due to the low coverage chosen for RIL sequencing, a sliding window approach was applied to evaluate a group of consecutive SNPs for genotyping [42]. The genotypes of all chromosomes of the 212 RILs were aligned and compared for the minimal of 100-kb intervals and the adjacent 100-kb intervals with the same genotype across the entire RIL population were recognized as a single recombination bin [42]. From bin markers, linkage groups were constructed using JoinMap v5.0 [29], selecting LOD scores from 2 to 10 to identify groups and the regression algorithm to perform ordering within each LG. The final linkage map was drawn using the R package LinkageMapView. QTL mapping was performed using MapQTL v6.0 [30], by selecting multiple QTL mapping (MQM) to detect potential QTLs with the LOD threshold of 2.5 in at least one environment.

## Supplementary information

**Additional file 1: Table S1.** The summary information of the sequencing and alignment results.

**Additional file 2: Figure S1.** The physical recombination map of 212 RILs on 20 chromosomes. Blue: the genotype of the resistant parent Zheng8903; Red: the genotype of the susceptible parent Yuhua4; Yellow: the heterozygous genotype; RILs were arranged from the top to the bottom and chromosomes were ranked from left to right.

**Additional file 3: Table S2.** Genotype and position of the bins in the peanut genetic linkage map obtained in this study. The letter A indicates the genotype of the parental line Zheng8903, and the letter B indicates the genotype of the parental line Yuhua4.

**Additional file 4: Table S3.** The phenotypes and KASP genotyping results of 47 lines. * stands for missing data.

## Data Availability

All data generated or analyzed during this study are included in the manuscript and its Additional file 1, Additional file 2, Additional file 3, and Additional file 4. The BAM files about resequencing data of RILs obtained in this study have been submitted to the BioProject database at NCBI under the BioProject ID: PRJNA602098. The materials used during the current study are available from the corresponding authors.

## Abbreviations

QTLs: Quantitative trait loci
RIL: Recombinant inbred line
LG: Linkage group
NBS-LRR: Nucleotide-binding site leucine-rich repeat
ORFs: Open reading frames
SNP: Single nucleotide polymorphisms
CC: Coiled-coil
TIR: Toll-interleukin receptor
NB-ARC: Nucleotide-binding domain shared with APAF-1, various R-proteins and CED-4
KASP: Kompetitive allele specific PCR
BAM: Binary alignment mapping
PVE: Phenotypic variance explained qWBR Qtl web blotch resistance
MAS: Marker assisted breeding
