# Supplementary material for: QTL mapping of web blotch resistance in peanut by high-throughput genome-wide sequencing
Source: BMC Plant Biol. 2020 Jun 3;20:249. doi: 10.1186/s12870-020-02455-8 (PMC7268717; doi:10.1186/s12870-020-02455-8)
Supplement: Supplementary file 2 — Additional file 2: Figure S1. The physical recombination map of 212 RILs on 20 chromosomes. Blue: the genotype of the resistant parent Zheng8903; Red: the genotype of the susceptible parent Yuhua4; Yellow: the heterozygous genotype; RILs were arranged from the top to the bottom and chromosomes were ranked from left to right. [file 12870_2020_2455_MOESM2_ESM.docx]

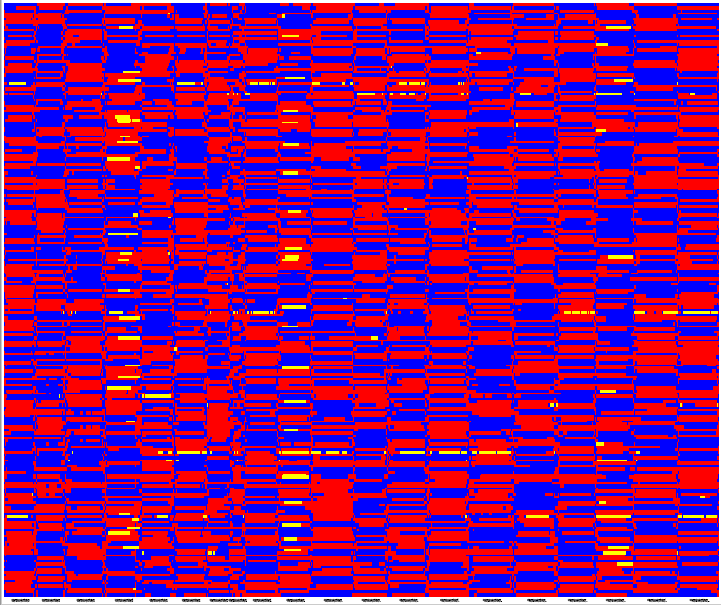


Supplementary Figure 1 The physical recombination map of 212 RILs on 20 chromosomes.

Blue: the genotype of the resistant parent Zheng8903; Red: the genotype of the susceptible parent Yuhua4; Yellow: the heterozygous genotype; RILs were arranged from the top to the bottom and chromosomes were ranked from left to right.
